# Mapping of inhomogeneous quasi-3D electrostatic field in electro-optic materials

**DOI:** 10.1038/s41598-021-81338-w

**Published:** 2021-01-25

**Authors:** Václav Dědič, Tomáš Fridrišek, Jan Franc, Jan Kunc, Martin Rejhon, Utpal N. Roy, Ralph B. James

**Affiliations:** 1 Faculty of Mathematics and Physics, Charles University, Ke Karlovu 3, 12116 Prague, Czech Republic; 2 Nonproliferation and National Security Department, Brookhaven National Laboratory, Upton, NY 11973 USA; 3 Savannah River National Laboratory, Aiken, SC 29808 USA

**Keywords:** Imaging and sensing, Sensors and biosensors, Materials science

## Abstract

This paper describes a new method for direct measurement and evaluation of the inhomogeneous electrostatic vector field with translational symmetry in electro-optic materials exhibiting the Pockels effect. It is based on the evaluation of maximum transmittance of low intensity light passing through a sample under a voltage bias. Here, the sample is located between rotating crossed polarizers, and camera images are obtained at each point to determine the electric field. The evaluation procedure is demonstrated using data acquired on a CdZnTeSe quasi-hemispheric semiconductor gamma-ray detector. In addition to CdTe-related compounds, the method can be used for various other materials showing $\overline{4}3m$ symmetry such as GaAs, CdTe, GaP, 3C-SiC, and ZnS. Furthermore, it can be generalized to other crystalline materials showing the Pockels effect. The method can be used to probe the space charge and the electric field in several kinds of electronic components and devices, as well as provide useful data on the role of defects, contact configurations and other surface and bulk inhomogeneities in the material that can affect the distribution of the internal electric field.

## Introduction

The electro-optic effect in $\overline{4}3m$ crystals has been studied theoretically, and the results have been published in several papers i.e.^1–4^. One of the common applications of past experiments has been the determination of the internal electric field in detectors and other electronic devices. Point electro-optic 1D electric field probes have been developed and demonstrated for scanning the quality of organic photovoltaics films^5^. We also highlight R&D investigations on real-time sensors of radiofrequency fields during magnetic resonance imaging^6^ and, especially, a point pigtailed vectorial sensor of intense electric fields^7^, which has become commercially available. Beside $\overline{4}3m$ crystals with a Pockels electro-optic coefficient typically of several pm/V^8^ and another widely used inorganic electrooptic material LiNbO_3_ with Pockels coefficient of ~ 30 pm/V^9,10^, there are other promising materials in the form of organic crystals and glasses with reported coefficients up to around 300 pm/V in near infrared range^11,12^, which potentially allow a significantly reduced electric field sensor volume while maintaining reasonable spatial resolution.

In our contribution, we show a new approach to perform high spatial resolution mapping of the internal quasi-3D electrostatic vector field with inhomogeneous distribution in a material showing a Pockels effect for an arbitrary crystallographic orientation. This generalized method is based on the transmittance measurements of the illuminated crystal placed between two rotating orthogonal polarizers. The transmittance distribution is monitored by a CMOS camera. The method is demonstrated here on a quasi-hemispheric CdZnTeSe detector. In principle, it could also be used to monitor an external electric field.

Detectors with hemispheric electrodes have been used for single charge collection^13^. An investigation of the electric field vector distribution measurement in quasi-hemispheric CdZnTeSe detectors is motivated by the need to understand the space charge accumulation in devices, which sometimes appears during semiconductor detector operation and can negatively affect detector performance. The electric fields in standard planar semi-insulating CdTe based detectors equipped with electrodes covering the entire opposite sides of the detector, in which case the direction of the electric field vector is expected to be more uniform, have already been studied by the Pockels effect using a cross-polarisers technique by several research groups^14–19^. Unlike the new method based on rotating polarizers, these simple traditional 1D methods use a fixed polarizer orientation and assume an electric field vector that is always perpendicular to the electrodes. Our recent reports show various applications of 1D electric field evaluation method related to the space-charge distribution and presence of deep defect levels in planar CdZnTe and CdZnTeSe detectors^20,21^. The effects of a high flux of incident X-rays, commonly used in medical applications, were also investigated^22^. Another application exploits the relatively high thermal neutron capture cross-section of ${ }^{113}\text{Cd}$ isotope in CdZnTe with the neutron reaction followed by effects accompanied with the creation of clouds of ionization charge. The effect can be used for the electro-optic high-flux neutron detection in the vicinity of nuclear reactors^23^. CdZnTeSe material was chosen for this study, because it has recently emerged as a promising material for X- and gamma-ray detectors due to its relatively low Te inclusions, absence from sub-grain boundary networks, and better compositional homogeneity compared to CdZnTe^24–26^, plus the 1D Pockels effect has been much less used in this material.

**Figure 1:**
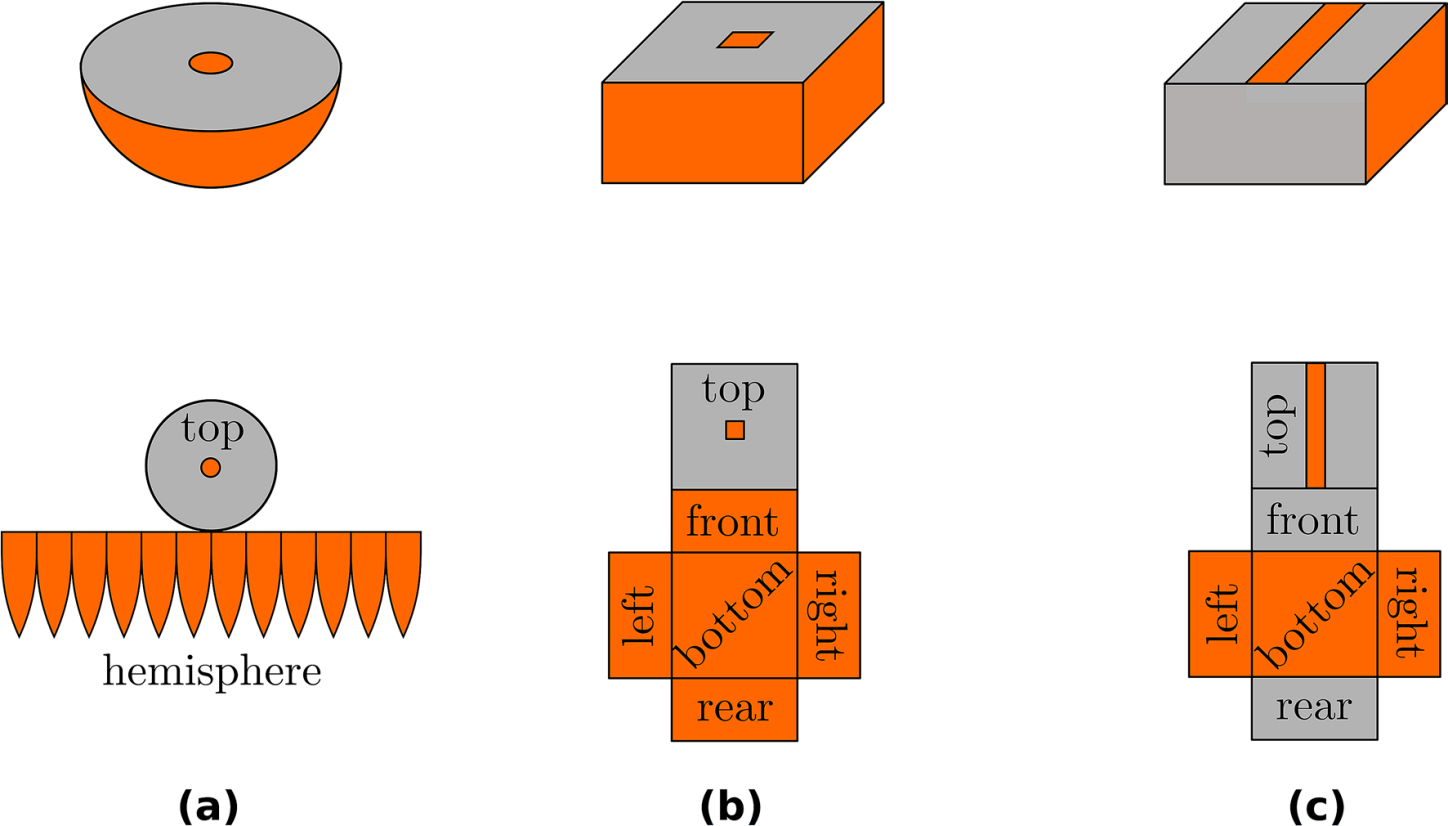
Pictorials (top) and net schemes (bottom) of ideal hemispheric detector (**a**), common quasi-hemispheric detector (**b**), and simplified quasi-hemispheric detector used in this study (**c**). Electrode areas are designated by orange color.

A pictorial of an ideal hemispheric detector is shown in Fig. 1a. Its rectangular modification is typically used for semiconductor detectors, because it is much easier for fabrication than curved surfaces (shown in Fig. 1b). We focus on a simplified case of a translationally symmetric quasi-hemispheric detector with a stripe electrode shown in Fig. 1c, which is suitable for demonstration of the new 3D cross-polarisers method. In this case, the electric field is constant along the stripe, and the quasi-3D electric field can be measured. Despite this dimensional reduction due to the additional symmetry, the method allows studies of the electric field as it would exist in the central section of a quasi-hemispheric detector (Fig. 1b). The spatial resolution of the method is given by the optical quality of the electro-optic crystal, camera zoom and pixel density and adjustment of the experimental setup. A space charge density distribution can be calculated from the measured electric field using Gauss law.

The main approach of the self-standing electrostatic vector field evaluation is based on a detailed derivation of a transmittance modulation with the crossed polarisers technique and an electro-optic crystal with arbitrary crystallographic orientation under an applied bias voltage.

## Theory

In this section, we show a step-by-step derivation of the so-called Pockels transmittance that is needed for reconstruction of the inhomogeneous electrostatic vector field in $\overline{4}3m$ crystals. The derivation is generally valid for all crystallographic and electric field vector orientations. A particular sample was used to demonstrate the validity of the approach for acquiring new information on internal electric fields and other useful parameters. The modeling approach is based on considerations of the wave retarder made of $\overline{4}3m$ crystals placed between two orthogonal polarizers in which the phase retardation is given by the Pockels effect. The derivation is adapted to a simultaneous rotation of both polarizers and for data processing using a computer due to the large scope and complexity required for calculating detailed electric field maps.

### Coordinate system

**Figure 2:**
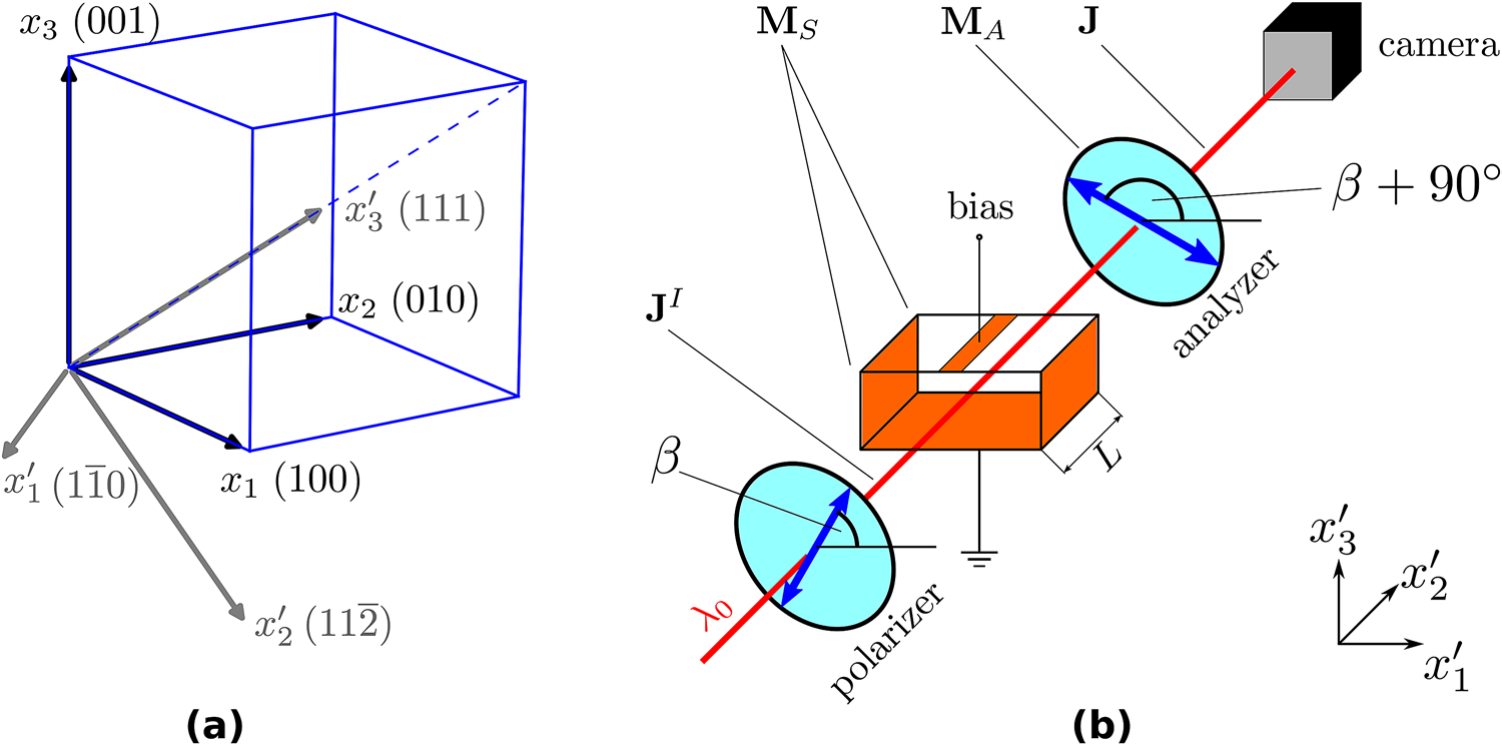
Non-dashed coordinate system (**a**) is formed by the main crystallographic axes (100), (010) and (001). The dashed coordinate system reflects the crystallographic orientation of the particular measured sample and the experimental arrangement. (**b**) shows the experimental arrangement for the cross-polarizers technique for a sample with a conductive stripe along the $x_{2}^{{\prime}} { }$ axis and full-area electrodes on opposite sides.

Two right-handed coordinate systems are used to describe the experimental arrangement, which depends on the particular crystallographic orientation of the measured sample (dashed system $x_{1}^{{\prime}} ,x_{2}^{{\prime}} ,x_{3}^{{\prime}}$) and the index ellipsoid important for derivation of the Pockels effect, which is defined in the main crystallographic axes^1^ (100), (010) and (001) (non-dashed coordinate system $x_{1} ,x_{2} ,x_{3}$, see Fig. 2a).

The vector variables can be transformed between the coordinate systems using the transformation matrix ${\mathbf{R}}$. The rows of ${\mathbf{R}}$ are formed by the unit vectors corresponding to the particular crystallographic orientations $\left( {1\overline{1}0} \right)$, $\left( {11\overline{2}} \right)$ and $\left( {111} \right)$ for the axes $x_{1}^{{\prime}}$, $x_{2}^{{\prime}}$ and $x_{3}^{{\prime}}$, respectively. The transformation of the common vector variable ${\mathbf{v}}$ between the coordinate systems can be written as

1 $${\mathbf{v}} = {\mathbf{R}} \cdot {\mathbf{v^{\prime}}},$$

where

2 $${\mathbf{R}} = \left( {\begin{array}{*{20}c} {\frac{1}{\sqrt 2 }} & { - \frac{1}{\sqrt 2 }} & 0 \\ {} & {} & {} \\ {\frac{1}{\sqrt 6 }} & {\frac{1}{\sqrt 6 }} & { - \frac{2}{\sqrt 6 }} \\ {} & {} & {} \\ {\frac{1}{\sqrt 3 }} & {\frac{1}{\sqrt 3 }} & {\frac{1}{\sqrt 3 }} \\ \end{array} } \right)$$

### Cross polarizers technique

The cross-polarizers technique is based on the biased sample acting as a dynamic electric-field-controlled wave retarder placed between two crossed polarisers (Fig. 2b). Low-intensity quasi-monochromatic light with the wavelength $\lambda_{0}$ propagates along the $x_{2}^{{\prime}}$ axis, and it passes through the polarizer forming a linearly polarised incident beam, then trough the biased sample and next through the second polarizer (analyzer). The transmittance distribution $T\left( {x_{1}^{{\prime}} ,x_{3}^{{\prime}} } \right)$ of the output beam is monitored by an InGaAs CMOS camera.

The transmittance $T$ in a particular camera pixel is derived using the Jones calculus^27^.The Jones vector ${\mathbf{J}}^{I}$ of the light beam that passes the polarizer can be written as

3 $${\mathbf{J}}^{I} = \left( {\begin{array}{*{20}l} {J_{1}^{I} } \hfill \\ {J_{3}^{I} } \hfill \\ \end{array} } \right) = \left( {\begin{array}{*{20}l} {{\text{cos}}\beta } \hfill \\ {{\text{sin}}\beta } \hfill \\ \end{array} } \right),$$

where the so-called polarizer angle $\beta$ is the angle between axis of transmittance of the polarizer and the $x_{1}^{{\prime}}$ axis (Fig. 2b).

Without any loss of generality, the Jones matrix ${\mathbf{M}}_{S}$ of the wave retarder (i.e., sample) with the fast axis forming the angle $\theta$ from $x_{1}^{{\prime}}$ axis in the $x_{1}^{{\prime}} x_{3}^{{\prime}}$ plane is given by

4 $${\mathbf{M}}_{S} = \left( {\begin{array}{*{20}l} {{\text{cos}}\theta } \hfill & { - {\text{sin}}\theta } \hfill \\ {{\text{sin}}\theta } \hfill & {{\text{cos}}\theta } \hfill \\ \end{array} } \right)\left( {\begin{array}{*{20}l} 1 \hfill & 0 \hfill \\ 0 \hfill & {{\text{e}}^{{ - {\mathfrak{i}}{\Gamma }\left( {\mathbf{E}} \right)}} } \hfill \\ \end{array} } \right)\left( {\begin{array}{*{20}l} {{\text{cos}}\theta } \hfill & {{\text{sin}}\theta } \hfill \\ { - {\text{sin}}\theta } \hfill & {{\text{cos}}\theta } \hfill \\ \end{array} } \right),$$

where

5 $${\Gamma }\left( {\mathbf{E}} \right) = \frac{2\pi }{{\lambda_{0} }}\left[ {n_{slow} \left( {\mathbf{E}} \right) - n_{fast} \left( {\mathbf{E}} \right)} \right]L$$

is the electric field dependent mutual phase shift between fast and slow axes. Here, $n_{slow} \left( {\mathbf{E}} \right) - n_{fast} \left( {\mathbf{E}} \right)$ is the difference of refractive indexes along the fast and slow axes of the electro-optic medium, respectively. $L$ is the path length through the sample.

${\mathbf{M}}_{A}$ is the Jones matrix of the analyzer with axis of transmittance perpendicular to the polarizer:

6 $${\mathbf{M}}_{A} = \left( {\begin{array}{*{20}l} {{\text{ cos}}^{2} \left( {\beta + \frac{\pi }{2}} \right)} \hfill & {{\text{sin}}\left( {\beta + \frac{\pi }{2}} \right){\text{cos}}\left( {\beta + \frac{\pi }{2}} \right)} \hfill \\ {} \hfill & {} \hfill \\ {{\text{sin}}\left( {\beta + \frac{\pi }{2}} \right){\text{cos}}\left( {\beta + \frac{\pi }{2}} \right)} \hfill & {{\text{ sin}}^{2} \left( {\beta + \frac{\pi }{2}} \right)} \hfill \\ \end{array} } \right).$$

Finally, the Jones vector of the output beam is

7 $${\mathbf{J}} = {\mathbf{M}}_{A} \cdot {\mathbf{M}}_{S} \cdot {\mathbf{J}}^{I} ,$$

and the transmittance $T\left( {\mathbf{E}} \right)$ of the whole system can be written as

8 $$T\left( {\mathbf{E}} \right) = {\mathbf{J}}^{{\text{T}}} \cdot {\mathbf{J}}^{*} = {\text{sin}}^{2} \frac{{{\Gamma }\left( {\mathbf{E}} \right)}}{2} \cdot {\text{sin}}^{2} \left[ {2\left( {\beta - \theta } \right)} \right].$$

From Eq. (8) it follows that the maximum of the transmittance $T_{max}$ for a given ${\Gamma }$ is

9 $$T_{max} \left( {\mathbf{E}} \right) = {\text{sin}}^{2} \frac{{{\Gamma }\left( {\mathbf{E}} \right)}}{2}.$$

It occurs when

10 $$\beta = \beta_{max} = \theta + \frac{\pi }{4}\left( {2k + 1} \right),\quad k \in {\mathbb{Z}}.$$

**Figure 3:**
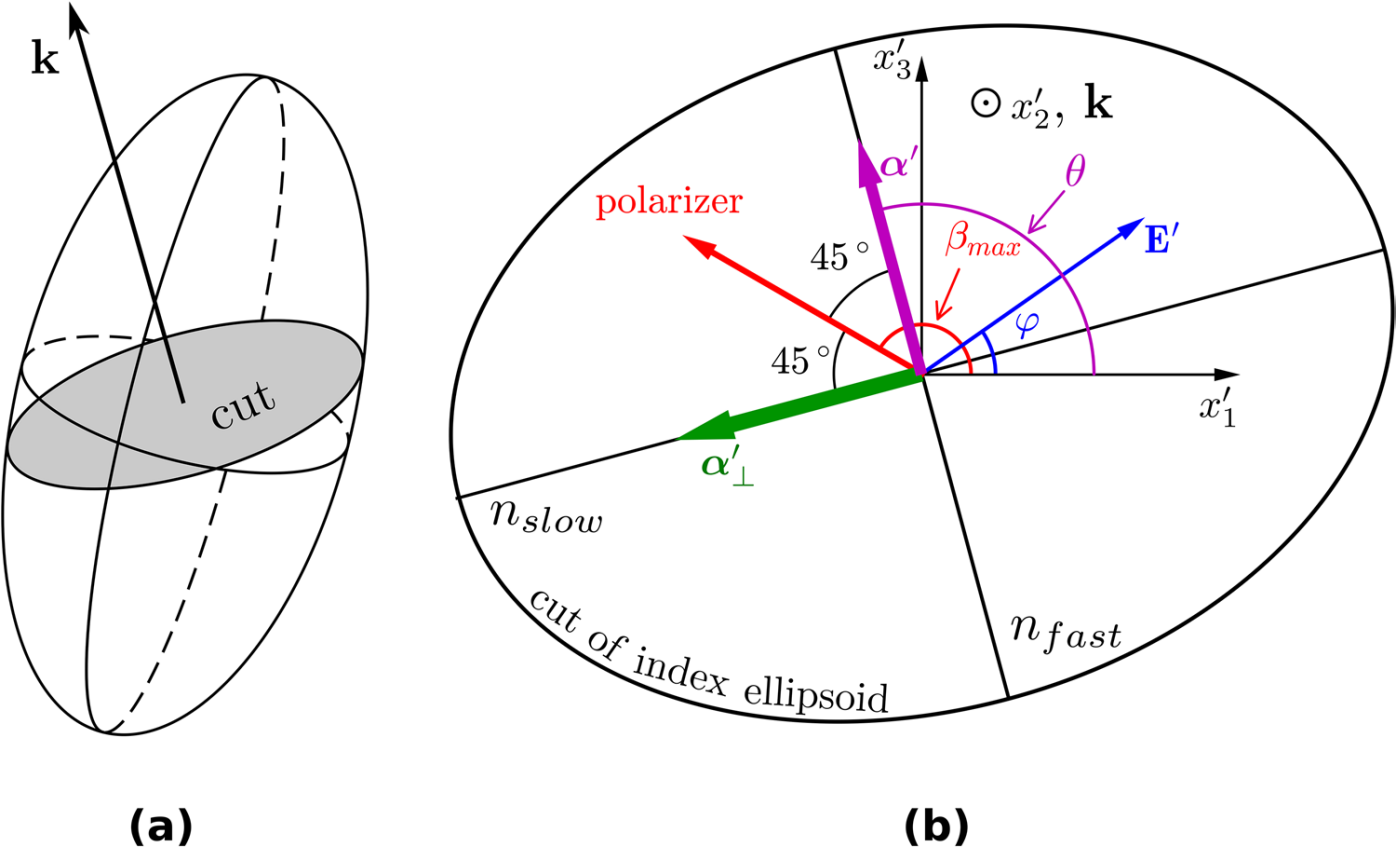
Index ellipsoid (**a**) and detail of its elliptical cut perpendicular to the direction of propagation of the testing light (**b**) represented by its ${\mathbf{k}}$-vector. The lengths of minor and major semi-axes correspond to $n_{fast}$ and $n_{slow}$, respectively.

In our geometry, $T_{max} \left( {\mathbf{E}} \right)$ occurs when the angle between the transmittance axes of the polarizers $\beta$ and axes of the retarder $\theta$ equals 45°. A schematic of the angles $\beta_{max}$ and $\theta$ and retarder axes is shown on the elliptical cut of the index ellipsoid in shown by Fig. 3.

### Pockels effect

For a coordinate system formed by the main crystallographic axes, the index ellipsoid of an electrooptic crystal with $\overline{4}3m$ symmetry has the following form^1^

11 $$\frac{{x_{1}^{2} + x_{2}^{2} + x_{3}^{2} }}{{n_{0}^{2} }} + 2r_{41} \left( {E_{1} x_{2} x_{3} + E_{2} x_{3} x_{1} + E_{3} x_{1} x_{2} } \right) = 1,$$

where $r_{41}$ and $n_{0}$ are the Pockels coefficient and the refractive index of the material, respectively. $E_{1}$, $E_{2}$ and $E_{3}$ are the components of the electric field vector ${\mathbf{E}}$.

A small change of the refractive index ${\Delta }n = {\Delta }n\left( {{\varvec{\alpha}},{\mathbf{E}}} \right)$ for given direction of light polarization ${\varvec{\alpha}} = \left( {\alpha_{1} ,\alpha_{2} ,\alpha_{3} } \right)$ and ${\mathbf{E}}$ can be derived from Eq. (11) using the substitution $x_{i} = \left( {n_{0} + {\Delta }n\left( {\mathbf{E}} \right)} \right)\alpha_{i}$:

12 $${\Delta }n\left( {\mathbf{E}} \right) \approx - n_{0}^{3} r_{41} E\left( {d_{1} \alpha_{2} \alpha_{3} + d_{2} \alpha_{3} \alpha_{1} + d_{3} \alpha_{1} \alpha_{2} } \right),$$

where ${\mathbf{d}} = \left( {d_{1} ,d_{2} ,d_{3} } \right)$ is the unit directional vector of the electric field ${\mathbf{E}} = E{\mathbf{d}}$, $\left| \alpha \right| = 1$, and small values of $({\Delta }n)^{2}$ and $r_{41} {\Delta }n\left( {\mathbf{E}} \right)$ are neglected.

The variables related to the Pockels effect are valid for a certain pixel and optical path in the sample. In order to characterize the whole sample in plane $x_{1}^{{\prime}} x_{3}^{{\prime}}$, it is necessary to calculate the Pockels effect separately at each camera pixel covering the sample to get the electric field vector distribution ${\mathbf{E}}\left( {x_{1}^{{\prime}} ,x_{3}^{{\prime}} } \right)$.

The light in a biased electrooptic material propagates in a form of two perpendicular linearly polarized modes with polarizations described by unit vectors ${\varvec{\alpha^{\prime}}}$ and ${\varvec{\alpha^{\prime}}_{ \bot }}$ whose directions are given by the semi-axes of the elliptical cut from Fig. 3 and with refractive indices $n\left( {\mathbf{E}} \right)$ and $n_{ \bot } \left( {\mathbf{E}} \right)$, respectively, that are subjects of the rule

13 $$\left| {n\left( {\mathbf{E}} \right) - n_{ \bot } \left( {\mathbf{E}} \right)} \right| = n_{slow} \left( {\mathbf{E}} \right) - n_{fast} \left( {\mathbf{E}} \right).$$

Small changes of these refractive indices ${\Delta }n\left( {\mathbf{E}} \right)$ and ${\Delta }n_{ \bot } \left( {\mathbf{E}} \right)$ are given by Eq. (12)

14 $$\begin{aligned} & {\Delta }n\left( {\mathbf{E}} \right) \approx - n_{0}^{3} r_{41} E\left( {d_{1} \alpha_{2} \alpha_{3} + d_{2} \alpha_{3} \alpha_{1} + d_{3} \alpha_{1} \alpha_{2} } \right), \\ & {\Delta }n_{ \bot } \left( {\mathbf{E}} \right) \approx - n_{0}^{3} r_{41} E\left( {d_{1} \alpha_{2 \bot } \alpha_{3 \bot } + d_{2} \alpha_{3 \bot } \alpha_{1 \bot } + d_{3} \alpha_{1 \bot } \alpha_{2 \bot } } \right), \\ \end{aligned}$$

respectively, where $\left( {\alpha_{1} ,\alpha_{2} ,\alpha_{3} } \right) = {\varvec{\alpha}} = {\mathbf{R}}^{ - 1} \cdot \user2{\alpha^{\prime}}$, $\left( {\alpha_{1 \bot } ,\alpha_{2 \bot } ,\alpha_{3 \bot } } \right) = {\varvec{\alpha}}_{ \bot } = {\mathbf{R}}^{ - 1} \cdot \user2{\alpha^{\prime}}_{ \bot }$ and $E{\mathbf{d}} = E{\mathbf{R}}^{ - 1} \cdot {\mathbf{d^{\prime}}} = {\mathbf{R}}^{ - 1} \cdot {\mathbf{E^{\prime}}}$.

If the electric field vector direction angle is $\varphi$ (see Fig. 3b), and polarizer angle is ${ }\beta_{max}$ from Eq. (10), then

$${\mathbf{E^{\prime}}} = E{\mathbf{d^{\prime}}} = E\left( {{\text{cos}}\varphi ,0,{\text{sin}}\varphi } \right),$$

15 $${\varvec{\alpha}}^{{\prime }} = \left( {\begin{array}{*{20}c} {{\text{cos}}\left( {{ }\beta_{max} - \pi /4} \right)} \\ 0 \\ {{\text{sin}}\left( {{ }\beta_{max} - \pi /4} \right)} \\ \end{array} } \right)\quad {\text{and}}\quad {\varvec{\alpha}}_{ \bot }^{{\prime }} = \left( {\begin{array}{*{20}c} {{\text{cos}}\left( {{ }\beta_{max} + \pi /4} \right)} \\ 0 \\ {{\text{sin}}\left( {{ }\beta_{max} + \pi /4} \right)} \\ \end{array} } \right).$$

Using Eqs. (13) and (14) in Eq. (5), we get the mutual phase shift ${\Gamma }\left( {\mathbf{E}} \right)$ as a function of the electric field magnitude $E$ and direction ${\mathbf{d}}$:

16 $${\Gamma }\left( {\mathbf{E}} \right) = \frac{2\pi }{{\lambda_{0} }}n_{0}^{3} r_{41} LE\left| {d_{1} \left( {\alpha_{2} \alpha_{3} - \alpha_{2 \bot } \alpha_{3 \bot } } \right) + d_{2} \left( {\alpha_{3} \alpha_{1} - \alpha_{3 \bot } \alpha_{1 \bot } } \right) + d_{3} \left( {\alpha_{1} \alpha_{2} - \alpha_{1 \bot } \alpha_{2 \bot } } \right)} \right|.$$

Combining Eqs. (9), (15) and (16) with respect to the transformation to the non-dashed coordinate system according to Eq. (1), the maximum of so-called Pockels transmittance $T_{max}$ for a certain point of the studied sample in the $x^{\prime}_{1} x^{\prime}_{3}$ plane is

17 $$T_{max} \left( {E,\varphi } \right) = {\text{sin}}^{2} \left( {\frac{\pi }{{\lambda_{0} }}n_{0}^{3} r_{41} LE\left| {\frac{{\cos \left( {2{ }\beta_{max} - \varphi } \right) - 5\cos \left( {2{ }\beta_{max} + \varphi } \right)}}{4\sqrt 3 }} \right|} \right).{ }$$

Here, $T_{max}$ at the given crystallographic orientation $\left( {1\overline{1}0} \right)$, $\left( {11\overline{2}} \right)$ and $\left( {111} \right)$ and for given experimental arrangement is a function of the electric field magnitude $E$ and its directional angle $\varphi$. In the case of maximum transmittance $T_{max}$ (see Eq. 9), the angle between polarizer $\beta_{max}$ and semi-axes $\theta$ of the ellipse from Fig. 3b should be $45^{ \circ }$.

**Figure 4:**
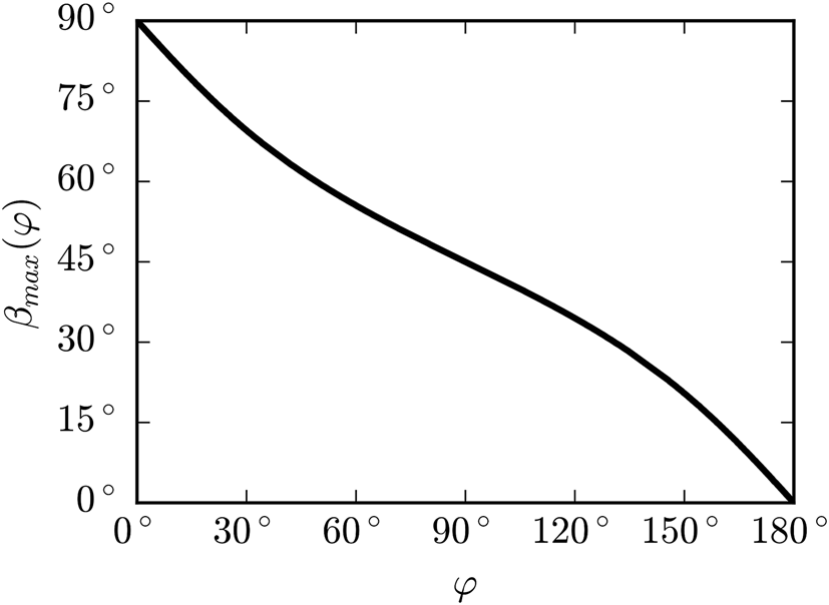
Calculated dependence of the polarizer angle $\beta_{max}$ for a given electric field directional vector $\varphi$ for crystallographic orientations $\left( {1\overline{1}0} \right)$, $\left( {11\overline{2}} \right)$ and $\left( {111} \right)$ and for the experimental arrangement used in this study.

The relation between polarizer angle $\beta_{max}$ at maximum Pockels transmittance $T_{max}$ and electric field directional angle $\varphi$ was calculated numerically from Eq. (17) for the fixed value $E$ , and the results are shown in Fig. 4. Extension of $\varphi$ to the whole interval of $0 - 360^{ \circ }$ can be easily made based on the knowledge of the polarity of sample electrodes. The 90°-periodicity of the Pockels transmittance on polarizer angle is apparent from Eq. (17).

The main idea of the evaluation of the electric field vector distribution ${\mathbf{E}}\left( {x_{1}^{{\prime}} ,x_{3}^{{\prime}} } \right)$ is based on finding the distributions of $T_{max} \left( {x_{1}^{{\prime}} ,x_{3}^{{\prime}} } \right)$ and $\beta_{max} \left( {x_{1}^{{\prime}} ,x_{3}^{{\prime}} } \right)$ from camera images taken at different polarizer angles $\beta$ and assigning the corresponding values of $\varphi \left( {x_{1}^{{\prime}} ,x_{3}^{{\prime}} } \right)$ and $E\left( {x_{1}^{{\prime}} ,x_{3}^{{\prime}} } \right)$ using data from Fig. 4 and inversion of Eq. (17), respectively.

## Results and discussion

### Transmittance measurement and electric-field evaluation

**Figure 5:**
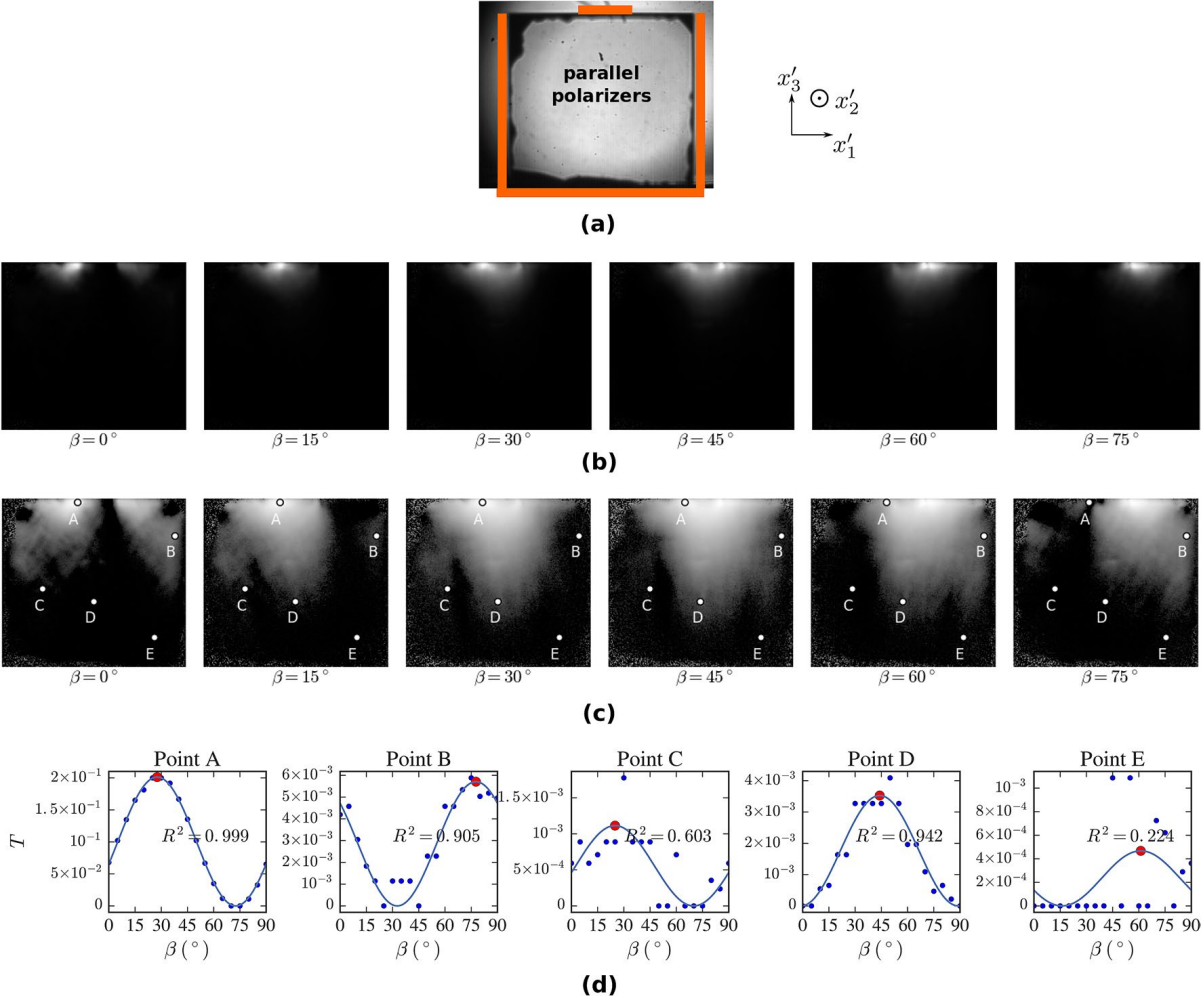
Camera image of the sample at zero bias and parallel polarizers illuminated with light at 1550 nm (**a**). Orange lines denote the positions of the electrodes. The sample is transparent except for several dark areas at the periphery, which are a sign of bad optical quality due to the surface finishing. Part (**b**) shows the Pockels transmittance $T\left( {x_{1}^{{\prime}} ,x_{3}^{{\prime}} } \right)$ of the biased sample (− 500 V on the stripe electrode, sample area only) at several polarizer angles $\beta$ in the range of 0°–90°. The $log_{10} T\left( {x_{1}^{{\prime}} ,x_{3}^{{\prime}} } \right)$ is shown in (**c**) including the positions of pixels A–E chosen for the demonstration of the analysis, in which case the $\left[ {\beta_{max} ,T_{max} } \right]$ positions (large red circles) (**d**) are found by fitting the experimental data (small circles) using Eq. (18) (solid curves).

**Figure 6:**
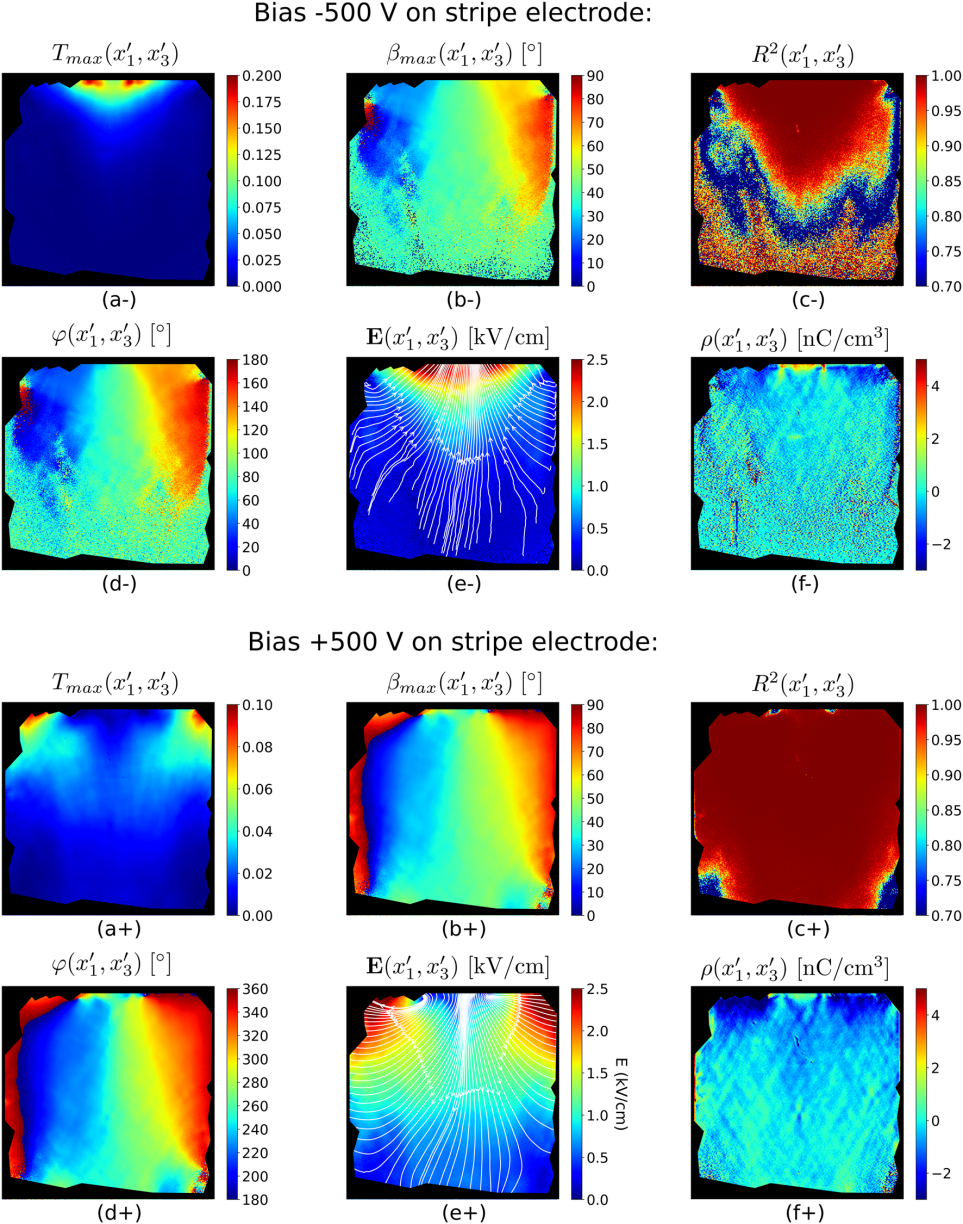
Electric field vector evaluation. Signs “ + ” and “−” indicate the polarity of the stripe electrode. Distributions of the maxima of Pockels transmittance $T_{max} \left( {x_{1}^{{\prime}} ,x_{3}^{{\prime}} } \right)$ (**a**) and corresponding polarizer angle $\beta_{max} \left( {x_{1}^{{\prime}} ,x_{3}^{{\prime}} } \right)$ (**b**) acquired by fitting the Pockels transmittance images using Eq. (18). Distribution of the coefficient of determination $R^{2} \left( {x_{1}^{{\prime}} ,x_{3}^{{\prime}} } \right)$ of the fitting is shown in (**c**). Electric field directional angle $\varphi$ distribution (**d**) was determined from $\beta_{max}$ based on the relation from Fig. 4. Electric field magnitude and direction distribution are shown in (**e**). Space charge distribution $\rho \left( {x_{1}^{{\prime}} ,x_{3}^{{\prime}} } \right)$ was calculated using Gauss law (**f**). Black areas denote the damaged parts along the edges of the sample.

The electric-field evaluation procedure (Fig. 5) is demonstrated for the case when the stripe electrode is biased to -500 V. The results are shown for both polarities (see Fig. 6). The procedure was executed on each pixel of the Pockels transmittance images covering the measured sample.

The biased sample was placed between two crossed polarizers (polarizer and analyzer, see Fig. 2b). Both the polarizer and the analyzer were mounted in the rotation mounts. The source of the low-intensity testing light was a light-emitting diode (LED) with a central wavelength at $\lambda_{0} = 1550\;\text{nm}$. The light beam was sufficiently collimated for the purposes of this experiment. CdZnTeSe is transparent at this wavelength, and the crystal does not show any significant photoconductivity at $\lambda_{0}$. The distribution of light transmittance $T\left( {x_{1}^{{\prime}} ,x_{3}^{{\prime}} } \right)$ of the system was monitored by an infrared InGaAs CMOS camera equipped with a zoom lens. The Pockels transmittance $T$ was then analyzed separately at each pixel of the camera image covering the measured sample. Each camera pixel represents an area of $30 \times 30 \;\mu {\text{m}}^{2}$ for the experimental arrangement used in this study.

The surfaces of the CdZnTeSe sample with dimensions of $7 \times 7 \times 7\;\text{mm}^3$ were optically polished. The electrode material is gold. The stripe electrode is 1 mm wide. For a wavelength at 1550 nm, the refractive index of the sample material is $n_{0} = 2.74$, and the electro-optic coefficient is $r_{41} = 6.7\;\text{pm/V}$.

Figure 5a shows a camera image of the measured sample placed between parallel polarizers at 0 V. The positions of electrodes are highlighted. The dark areas at the sample periphery are related to the damaged edges formed during the sample preparation. Besides these few areas, which can not be considered for further analysis, the transmittance of the sample is relatively homogeneous for the incident light at 1550 nm.

The polariser and the analyzer were kept crossed during the electric-field measurement. Both were rotated with a step of $5^{ \circ }$ in the range of 0°–90°, and a camera image was acquired at each step. Figure 5b shows the transmittance distributions $T\left( {x_{1}^{{\prime}} ,x_{3}^{{\prime}} } \right)$ of the biased sample at several polarizer angles $\beta$ when the stripe electrode was biased at − 500 V. $T\left( {x_{1}^{{\prime}} ,x_{3}^{{\prime}} } \right)$ is calculated as a ratio of the camera images representing the monitored light intensities of the biased sample placed between crossed polarisers and the unbiased sample with parallel polarizers. $T\left( {x_{1}^{{\prime}} ,x_{3}^{{\prime}} } \right)$ varies between 0 and 1. The camera thermal noise was taken into account. Figure 5b shows thermal noise-free data from the sample area only. Significant changes of the Pockels transmittance at different polariser angles and different spots of the sample reflect the complex dependence of the transmittance on $E$ and $\varphi$ as described by Eq. (17).

In order to highlight the changes in transmittance, logarithms of the transmittance distributions are shown in Fig. 5c along with the positions of five camera pixels marked as A–E representing sample areas with different transmittance behavior. It is apparent from Eq. (17) and the condition from Eq. (10) that the Pockels transmittance $T$ depends on the squared trigonometric sine of the polarizer angle $\beta$ for a fixed electric field ${\text{E}}\left( {E = const.,\varphi = const.} \right)$. Figure 5d shows this dependence for pixels A–E with a step ${\Delta }\beta = 5^{ \circ }$. Experimental data (small circles) were fit using the function

18 $$T\left( \beta \right) = T_{max} {\text{sin}}^{2} \left[ {2\left( {\beta - \beta_{max} + \frac{\pi }{4}\left( {2k + 1} \right)} \right)} \right], \quad k \in {\mathbb{Z}}$$

in order to localize the maxima of Pockels transmittance $T_{max}$ and corresponding polarizer angle $\beta_{max}$ (large red circles). It is apparent that the fit works relatively well with a coefficient of determination $R^{2} > 0.9$ for the pixels with maximum transmittance ranging above approximately 0.002 (pixels A, B and D). On the other hand, $T\left( \beta \right)$ points at pixels C and E show a large dispersion due to a very low or zero electric field. The effect here is more than a hundred times lower than at pixel A.

The distributions of $T_{max} \left( {x_{1}^{{\prime}} ,x_{3}^{{\prime}} } \right)$ and $\beta_{max} \left( {x_{1}^{{\prime}} ,x_{3}^{{\prime}} } \right)$ shown in Fig. 6a−,b−, respectively, were found by fitting all the sample pixels according to Eq. (18). The corresponding distribution of the coefficient of determination $R^{2} \left( {x_{1}^{{\prime}} ,x_{3}^{{\prime}} } \right)$ is shown in Fig. 6c−. Here it is apparent that the fit fails for the pixels lying below the blue part of Fig. 6c−, where $R^{2}$ has unstable random values. In this part the Pockels transmittance is very low due to a negligible electric field.

Figure 6 shows the results of the electric-field measurements for both bias polarities. It is apparent from Fig. 6c+ that in this case, the fit works well over the whole sample area due to a significant electric field magnitude throughout the sample.

The electric field vector directional angle distribution $\varphi \left( {x_{1}^{{\prime}} ,x_{3}^{{\prime}} } \right)$ shown in Fig. 6d− is evaluated from $\beta_{max} \left( {x_{1}^{{\prime}} ,x_{3}^{{\prime}} } \right)$, which was calculated numerically with respect to the polarity of the electrodes (see Fig. 4).

The electric field magnitude distribution $E\left( {x_{1}^{{\prime}} ,x_{3}^{{\prime}} } \right)$ is calculated using an inversion of Eq. (17) and the measured distributions of $\beta_{max} \left( {x_{1}^{{\prime}} ,x_{3}^{{\prime}} } \right),{ }\varphi \left( {x_{1}^{{\prime}} ,x_{3}^{{\prime}} } \right)$ and $T_{max} \left( {x_{1}^{{\prime}} ,x_{3}^{{\prime}} } \right)$ for $\beta - \theta = \frac{\pi }{4}$. Thus the complete information about the electric field vector distribution ${\mathbf{E}}\left( {x_{1}^{{\prime}} ,x_{3}^{{\prime}} } \right)$ is obtained as shown in Fig. 6e−.

The space charge density distribution $\rho \left( {x_{1}^{{\prime}} ,x_{3}^{{\prime}} } \right)$ can be calculated from ${\mathbf{E}}\left( {x_{1}^{{\prime}} ,x_{3}^{{\prime}} } \right)$ using Gauss law

19 $$\nabla \cdot {\mathbf{E}}\left( {x_{1}^{{\prime}} ,x_{3}^{{\prime}} } \right) = \frac{{\partial E^{\prime}_{1} \left( {x_{1}^{{\prime}} ,x_{3}^{{\prime}} } \right)}}{{\partial x^{\prime}_{1} }} + \frac{{\partial E^{\prime}_{3} \left( {x_{1}^{{\prime}} ,x_{3}^{{\prime}} } \right)}}{{\partial x^{\prime}_{3} }} = \frac{{\rho \left( {x_{1}^{{\prime}} ,x_{3}^{{\prime}} } \right)}}{{\varepsilon_{0} }},$$

where $\varepsilon_{0}$ is the vacuum permittivity. Here $\rho \left( {x_{1}^{{\prime}} ,x_{3}^{{\prime}} } \right)$ is the total space charge density, which consists of the sum of free and bound charge densities. The space charge distribution evaluated from the experimental data is shown in Fig. 6f−.

**Figure 7:**
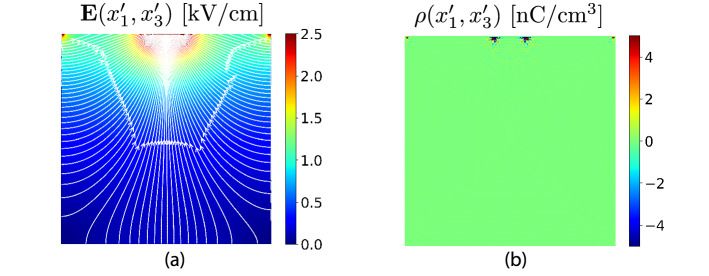
Simulated electric field vector distribution (**a**) for space charge free quasi-hemispheric detector. Space charge distribution (**b**) calculated from the electric field according the Eq. (19). Bias was set to − 500 V on the stripe electrode. For a reversed polarity, the electric field magnitudes remain the same, but the arrows representing the E-field directions flip into the opposite direction.

To validate the method, we performed a simulation of the electric field and the space charge distributions of an ideal quasi-hemispheric detector without any accumulated space charge (see Fig. 7). This electric field vector distribution was obtained by a numerical solution of the homogeneous Poisson equation. The Poisson equation was solved on a triangular grid by a finite element method. The average spacing of the solution nodes was 10 microns. The detector used in the simulation was of the same dimensions and electrode areas as the measured sample. Deviations of the measured electric field distributions shown in Fig. 6e from simulated data shown in Fig. 7a are related to the presence of charge carriers and trapped carriers at deep defect levels in the measured sample.

## Conclusions

A method for evaluating the quasi-3D electric field vector distribution in an electro-optic medium was derived and demonstrated on a quasi-hemispheric CdZnTeSe detector. The method is based on the electro-optic modulation of the transmittance in a biased crystal with $\overline{4}3m$ symmetry based on Pockels-effect data for a sample placed between two orthogonal polarizers that are simultaneously rotated. The distribution of the transmittance was monitored by a CMOS camera. From the crystallographic orientation of the studied crystal and the transmittance camera images, it is possible to reconstruct the electric field vector and the space charge density distributions. The spatial resolution of measured distributions was $30{ }\;\mu{\text{m}}$ in this experimental setup, but, in principle, it is limited by the diffraction limit. The proposed method can be generalized to a wide range of electro-optic materials with different crystallographic symmetries.

## Materials and methods

### Electro-optic crystal

The measured sample had dimensions of $7 \times 7 \times 7$ mm^3^; it was cut from a semi-insulating detector-grade Cd_0.9_Zn_0.1_Te_0.96_Se_0.04_ single crystal with $\overline{4}3m$ symmetry. The ingot was grown by the travelling heather method at Brookhaven National Laboratory^24,25^. The surfaces of the sample were optically polished at the Charles University. The gold electrodes were chemically deposited using a 1% AuCl_3_ water solution for 1 min. The stripe electrode was 1 mm wide. The crystallographic orientation of the sample was determined by the standard Laue method. The refractive index of the sample material is $n_{0} = 2.74$, and the electro-optic coefficient is $r_{41} = 6.7$ pm/V, both values shown for light at a wavelenght of 1550 nm.

### Cross polarizers experimental setup

The source of low-intensity testing light was a standard epoxy-encased LED at a central wavelength of $\lambda_{0} = 1550$ nm with a FWHM of 100 nm operating at a forward current of 100 mA (LED 1550E, *Thorlabs*). The LED was placed into the focus of a convex lens with a diameter of 54 mm and focal length of 100 mm. The measured sample was placed approximately 1 m from the LED. The incident light beam was sufficiently collimated for the purposes of the experiment. CdZnTeSe is transparent, and it did not show any significant photoconductivity at $\lambda_{0}$. The polariser and analyzer were near-infrared nanoparticle film linear polarizers with extinction ratios of $10^{8} :1$ at $\lambda_{0}$ (LPNIR100, *Thorlabs*). Both were placed in optical mounts equipped with an angular rotational capability with a precision of $\pm 0.5^{ \circ }$. The distribution of the light transmittance $T\left( {x_{1}^{{\prime}} ,x_{3}^{{\prime}} } \right)$ of the system was monitored by an infrared InGaAs CMOS camera Xenics Xeva with a resolution of $320 \times 256$ pixels equipped with a zoom lens. The temperature of the camera chip was stabilized at 245 K by an integrated 3-stage thermoelectric cooler in order to decrease the thermal noise. The sample was biased by a ISEG SHQ 122 M voltage source.
